# Three bamboo-feeding new species of the genus *Vekunta* Distant (Hemiptera, Fulgoromorpha, Derbidae) from Guizhou Province, China

**DOI:** 10.3897/zookeys.1289.186813

**Published:** 2026-08-14

**Authors:** Xing Yao, Yong-Jin Sui, Sha-Sha Lv, Jian-Kun Long, Zhi-Min Chang, Zi-Zhong Li, Xiang-Sheng Chen, Lin Yang

**Affiliations:** 1 Institute of Entomology, Guizhou University, Guiyang, Guizhou, 550025, China Guizhou Key Laboratory of Agricultural Biosecurity, Guizhou University Guiyang China; 2 The Provincial Special Key Laboratory for Development and Utilization of Insect Resources of Guizhou, Guizhou University, Guiyang, Guizhou, 550025, China Institute of Entomology, Guizhou University Guiyang China; 3 Guizhou Key Laboratory of Agricultural Biosecurity, Guizhou University, Guiyang, Guizhou, 550025, China The Provincial Special Key Laboratory for Development and Utilization of Insect Resources of Guizhou, Guizhou University Guiyang China

**Keywords:** Bamboo pest, derbid planthopper, Fulgoroidea, morphology, new taxa, taxonomy

## Abstract

Three bamboo-feeding new species of the genus *Vekunta* Distant, 1906, *V. deflecta* Yao, Sui & Chen, **sp. nov**., *V. fodingshanensis* Yao, Sui & Chen, **sp. nov**. and *V. furcata* Yao, Sui & Chen, **sp. nov**., are described and illustrated from Guizhou Province, China. *Vekunta fuscolineata*, previously known from Korea, is recorded from China for the first time. A checklist and a key to the males of the genus *Vekunta* from China are supplemented.

## Introduction

The planthopper family Derbidae Spinola, 1839 currently comprises three subfamilies divided into 22 tribes (Bourgoin 2026). Approximately nine tribes, 41 genera, and 184 species of Derbidae are known in China. Almost all members of the family in China occur in the Oriental region, especially in southern China.

The genus *Vekunta* Distant, 1906 belongs to the tribe Cenchreini Muir, 1918. It was first established as the genus *Temesa* by Melichar (1903) in the Achilidae Stål, 1866. Distant (1906a) found that *Temesa* Melichar, 1903 was invalid, as it is junior homonym of *Temesa* Adams, 1855 (Mollusca) and, therefore, proposed a replacement name, *Vekunta*. Distant (1906b) later designated *Temesa tenella* Melichar, 1903 as the type species. Kirkaldy (1907) proposed that the genus belongs to the family Derbidae, a classification that has been maintained to today. The genus has been studied by Melichar (1903, 1914), Bierman (1910), Muir (1913, 1914, 1915, 1917, 1922), Matsumura (1914, 1940), Schumacher (1915), Metcalf (1945), Fennah (1956), Yang and Wu (1994), Liang (2000), Wu and Liang (2001), Löcker et al. (2009), Rahman et al. (2012), and Sui and Chen (2019), among others. To date, it comprises 45 species distributed across the Australasian, Oriental, and Palaearctic regions. Twenty-nine species are known in China, with records in Taiwan (24 species), Guizhou (four species), Yunnan (three species), the Xizang Autonomous Region (one species), the Guangxi Zhuang Autonomous Region (one species), Zhejiang (one species), and Hunan (one species).

Here, three new bamboo-feeding species, *V. deflecta* Yao, Sui & Chen, sp. nov., *V. fodingshanensis* Yao, Sui & Chen, sp. nov. and *V. furcata* Yao, Sui & Chen, sp. nov. are described and illustrated from Guizhou Province, China. In addition, *V. fuscolineata* is recorded from China for the first time. A checklist and a key to the males of the genus from China are also provided.

## Materials and methods

The morphological terminology follows Bourgoin (1987), Bourgoin and Huang (1990), and Yang and Wu (1994). Body length was measured from apex of vertex to tip of forewing using the Keyence VHX-1000E system. Standard terminology of venation follows Bourgoin et al. (2015). All descriptions and illustrations are based on dried specimens. External morphology was observed under a stereoscopic microscope, and all measurements were taken with an ocular micrometer. Colour photographs of adult habitus were taken with the Keyence VHX-6000 system. Genital segments were detached and macerated in 10% NaOH solution, then transferred to glycerine jelly for drawing under a Leica MZ 12.5 stereomicroscope. The resulting line drawings were scanned with a Canon CanoScan LiDE 220 flat-bed scanner and imported into Adobe Photoshop CC for labelling and figure assembly. Dissected terminalia are preserved within glycerine in small plastic tubes, which are pinned together with their respective specimens.

The type specimens are deposited in the Institute of Entomology, Guizhou University, Guiyang, Guizhou Province, China (**GUGC**).

## Taxonomy

### Genus Vekunta

Taxon classificationAnimaliaHemipteraDerbidae

Distant, 1906

D717A028-036A-5752-9D59-CAD3C7EC3C37

Figs 1, 2, 3, 4, 5, 6, 7, 8, 9, 10, 11, 12, 13, 14, 15, 16, 17, 18, 19, 20, 21, 22, 23, 24, 25, 26, 27, 28, 29, 30, 31, 32, 33, 34, 35, 36, 37, 38, 39

Temesa Melichar, 1903: 40; preoccupied by Temesa (Mollusca) Adams, 1855.
Vekunta Distant, 1906a: 8—Distant 1906b: 287; Yang and Wu 1994: 97; Liang and Wu 2001: 511–512; Löcker et al. 2009: 15; Rahman et al. 2012: 24; Sui and Chen 2019: 57.

#### Type species.

*Temesa tenella* Melichar, 1903.

**Figures 1–5.**
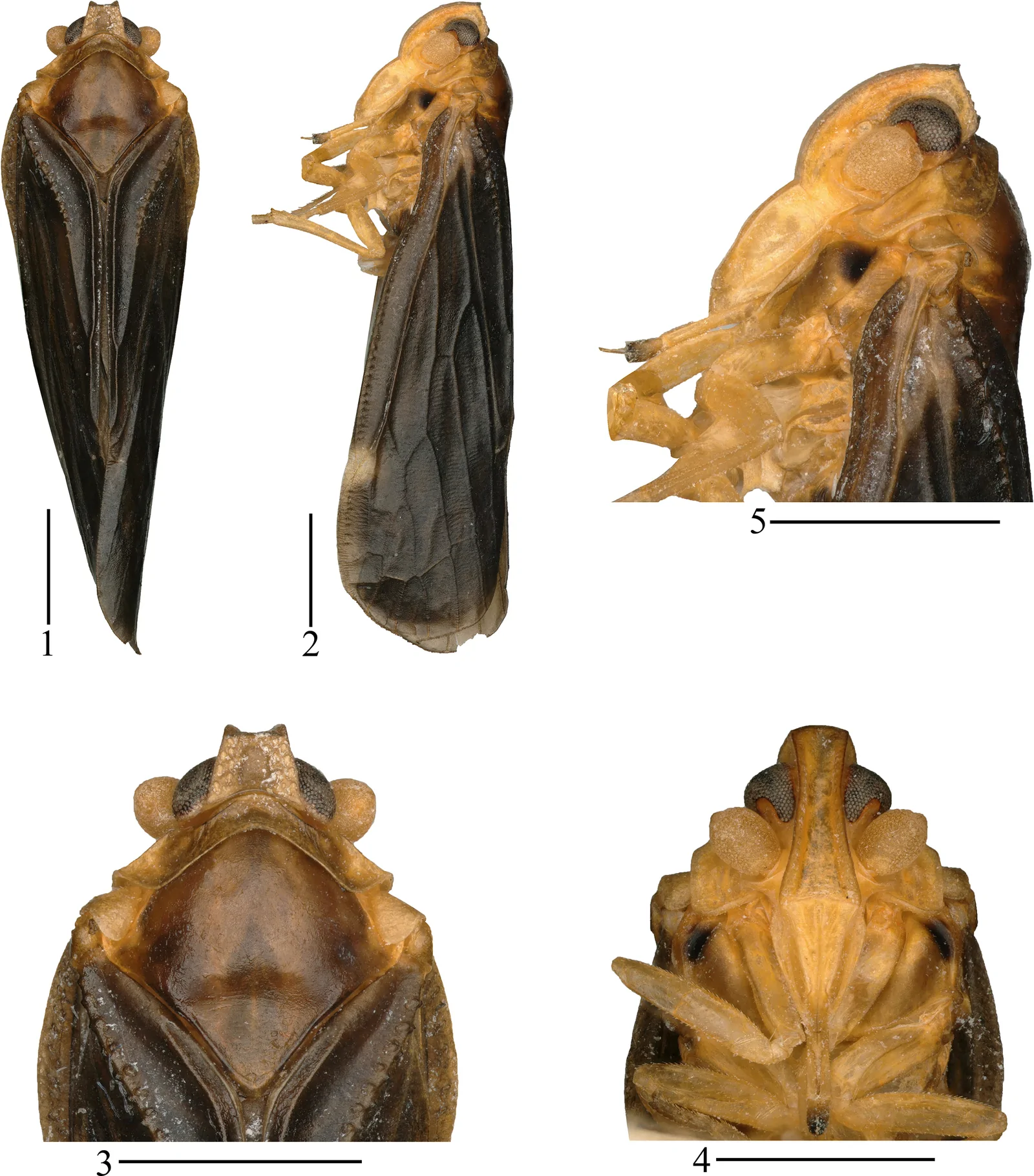
*Vekunta deflecta* Yao, Sui & Chen, sp. nov., male. **1**. Habitus, dorsal view; **2**. Habitus lateral view; **3**. Head and thorax, dorsal view; **4**. Face; **5**. Head and thorax, left lateral view. Scale bars: 1 mm.

**Figures 6–13.**
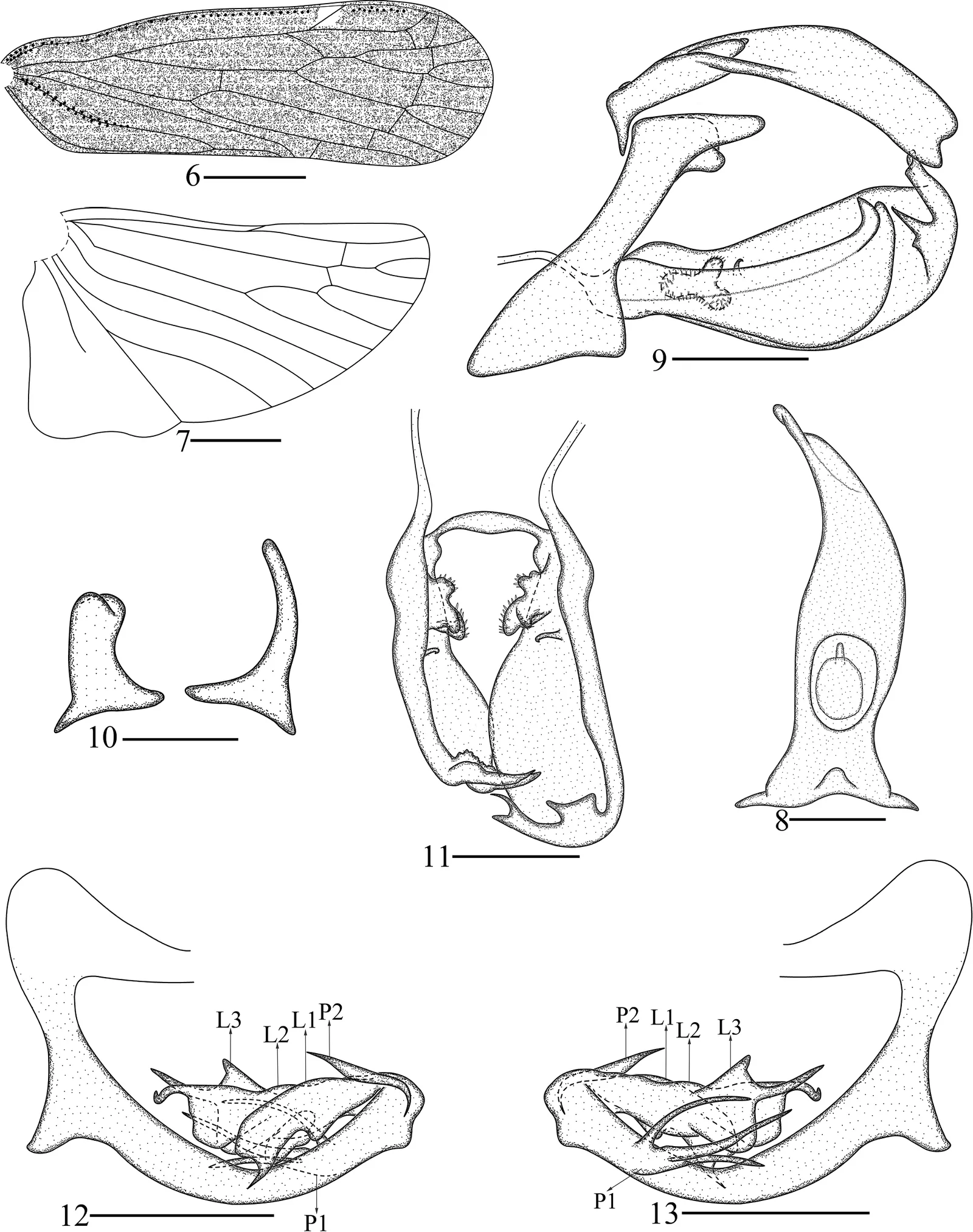
*Vekunta deflecta* Yao, Sui & Chen, sp. nov., male. **6**. Forewing; **7**. Hindwing; **8**. Anal tube, dorsal view; **9**. Terminalia, left lateral view; **10**. Dorsocaudal processes of pygofer, dorsal view; **11**. Gonostyli, dorsal view; **12**. Phallic complex, left lateral view; **13**. Phallic complex, right lateral view. Scale bars: 1 mm (**6, 7**); 0.5 mm (**9, 11–13**); 0.3 mm (**8, 10**).

**Figures 14–18.**
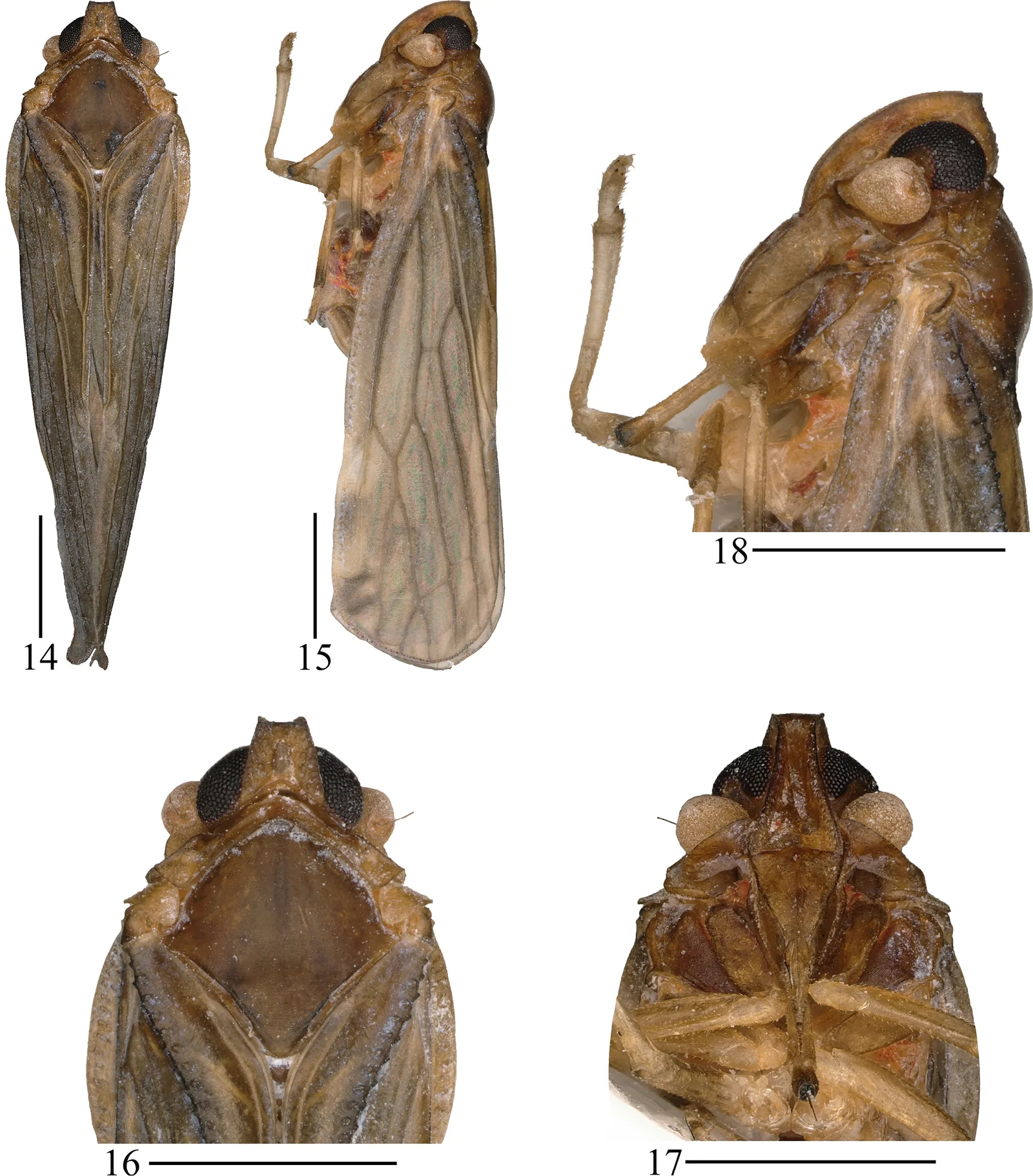
*Vekunta fodingshanensis* Yao, Sui & Chen, sp. nov., male. **14**. Habitus, dorsal view; **15**. Habitus lateral view; **16**. Head and thorax, dorsal view; **17**. Face; **18**. Head and thorax, left lateral view. Scale bars: 1 mm.

**Figures 19–26.**
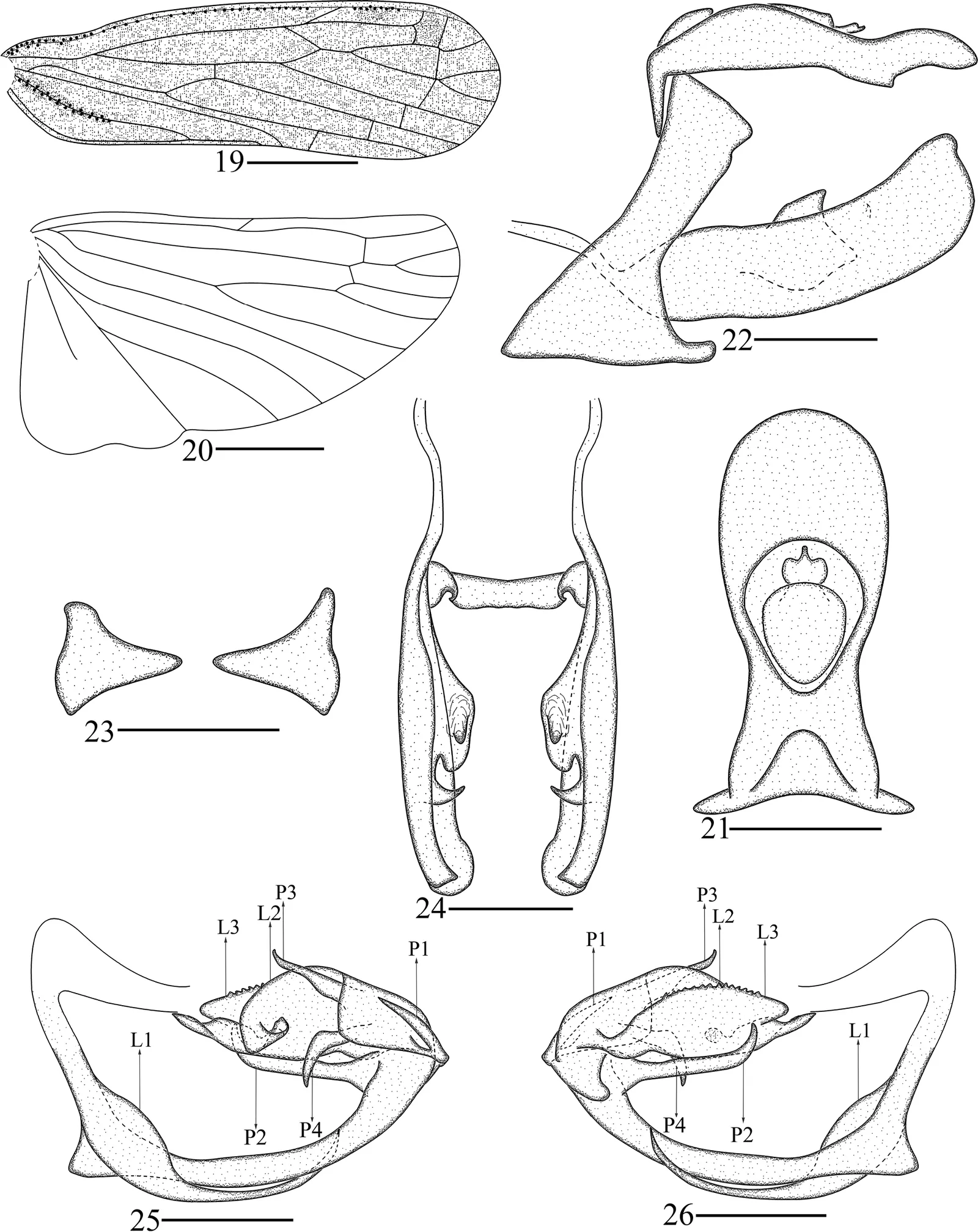
*Vekunta fodingshanensis* Yao, Sui & Chen, sp. nov., male. **19**. Forewing; **20**. Hindwing; **21**. Anal tube, dorsal view; **22**. Terminalia, left lateral view; **23**. Dorsocaudal processes of pygofer, dorsal view; **24**. Gonostyli, dorsal view; **25**. Phallic complex, left lateral view; **26**. Phallic complex, right lateral view. Scale bars: 1 mm (**19, 20**); 0.3 mm (**21–26**).

**Figures 27–31.**
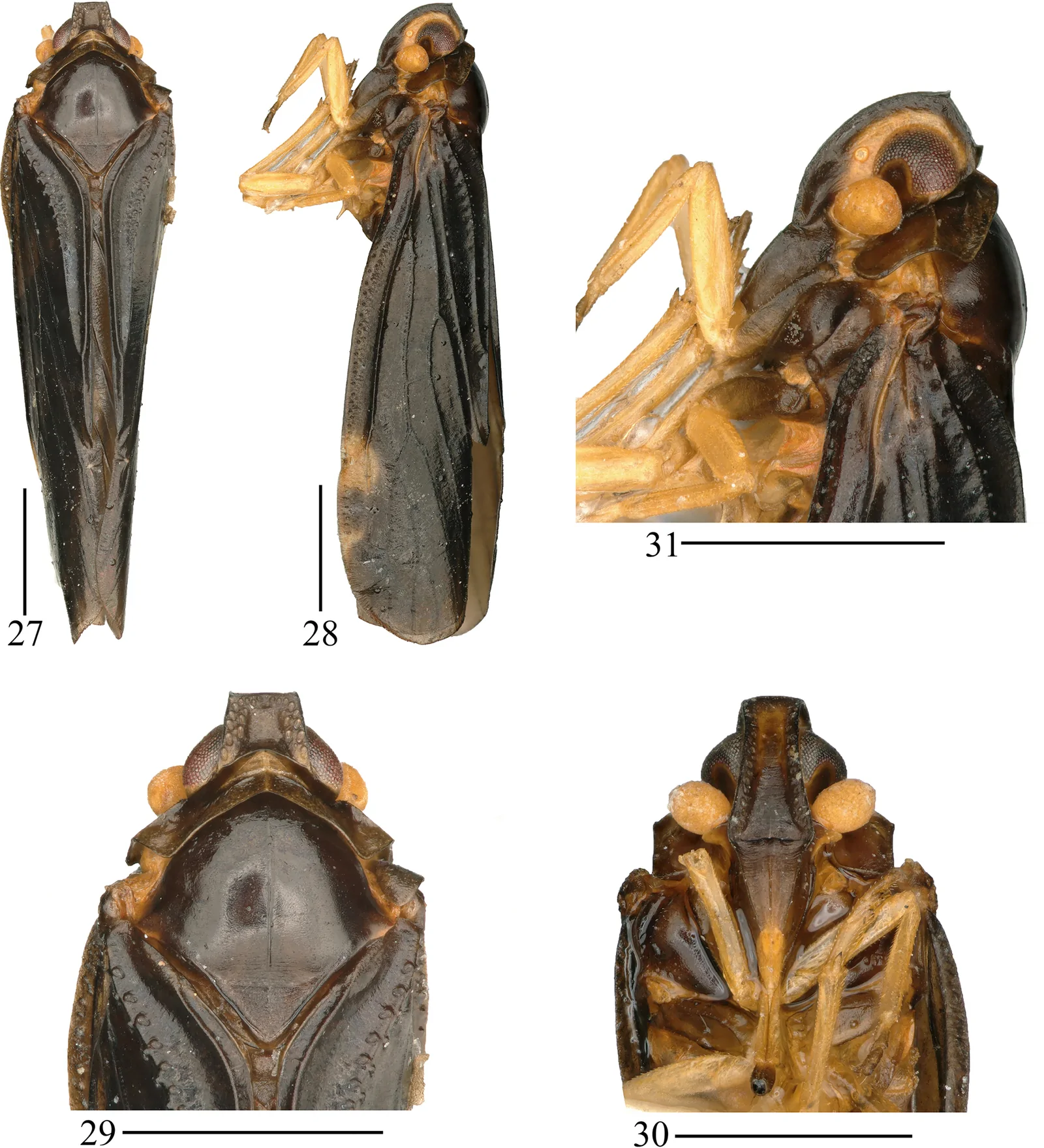
*Vekunta furcata* Yao, Sui & Chen, sp. nov., male. **27**. Habitus, dorsal view; **28**. Habitus lateral view; **29**. Head and thorax, dorsal view; **30**. Face; **31**. Head and thorax, left lateral view. Scale bars: 1 mm.

**Figures 32–39.**
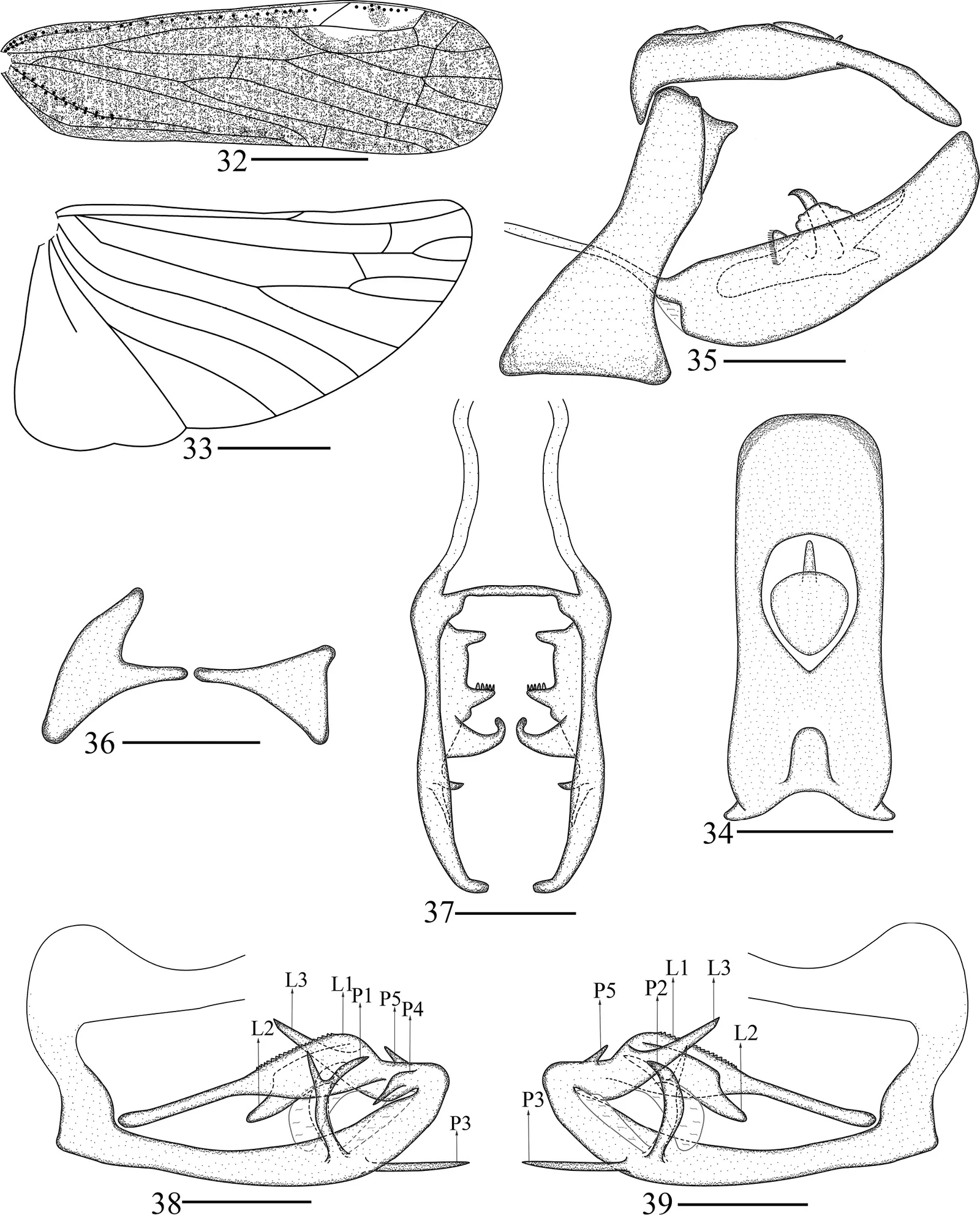
*Vekunta furcata* Yao, Sui & Chen, sp. nov., male. **32**. Forewing; **33**. Hindwing; **34**. Anal tube, dorsal view; **35**. Terminalia, left lateral view; **36**. Dorsocaudal processes of pygofer, dorsal view; **37**. Gonostyli, ventral view; **38**. Phallic complex, left lateral view; **39**. Phallic complex, right lateral view. Scale bars: 1 mm (**32, 33**); 0.3 mm (**34, 35, 37–39**); 0.2 mm (**36**).

#### Modified characters.

***Head*** (Figs 1–5, 14–18, 27–31) narrower than pronotum. ***Vertex*** (Figs 1, 3, 14, 16, 27, 29) trapezoidal, broader at base. ***Frons*** (Figs 4, 17, 30) rectangular; median carina absent; lateral margins distinctly separated. ***Second antennomere*** (Figs 1–5, 14–18, 27–31) oval. ***Subantennal process*** (Figs 4, 17, 30) small or absent. Ocelli (Figs 5, 18, 31) present. ***Forewing*** (Figs 6, 19, 32) with short subcostal cell; RP and MP_1+2_ usually fused for a distance; MP with two sectors; CuA bifurcated near basal third; costal margin and Pcu bearing tubercles; Pcu+A1 reaching forewing margin near midlength. ***Hindwing*** (Figs 7, 20, 33) slightly shorter than forewing; MP apically bifurcated; CuA with three terminals. CuP and Pcu unbranched. Hind tibia without lateral spine. Spinal formula of hind leg 7–6–6.

##### Checklist of species of *Vekunta* Distant, 1906 from China

*V. albipennis* Matsumura, 1914; China (Taiwan)

*V. asymmetrica* Liang & Wu, 2001; China (Xizang)

*V. atripennis* Matsumura, 1940; China (Taiwan)

*V. bambusana* Sui & Chen, 2019; China (Guangxi, Guizhou)

*V. botelensis* Matsumura, 1940; China (Taiwan)

*V. commendata* Yang & Wu, 1994; China (Taiwan)

*V. deflecta* Yao, Sui & Chen, sp. nov.; China (Guizhou)

*V. diluta* Yang & Wu, 1994; China (Taiwan)

*V. extima* Yang & Wu, 1994; China (Taiwan)

*V. fera* Yang & Wu, 1994; China (Taiwan)

*V. fodingshanensis* Yao, Sui & Chen, sp. nov.; China (Guizhou)

*V. furcata* Yao, Sui & Chen, sp. nov.; China (Guizhou)

*V. fuscolineata* Rahman, Kwon & Suh, 2012; China (Guizhou), Korea (new record for China)

*V. gracilenta* Yang & Wu, 1994; China (Taiwan)

*V. intermedia* Yang & Wu, 1994; China (Taiwan)

*V. kotoshonis* Matsumura, 1940; China (Taiwan)

*V. lyricen* Fennah, 1956; China (Taiwan)

*V. maculata* Matsumura, 1914; China (Taiwan)

*V. makii* Muir, 1914; China (Taiwan)

*V. malloti* Matsumura, 1914; China (Taiwan), Japan

*V. memoranda* Yang & Wu, 1994; China (Taiwan)

*V. nigra* Yang & Wu, 1994; China (Taiwan)

*V. nigrolineata* Muir, 1914; China (Taiwan)

*V. nivea* Fennah, 1956; China (Zhejiang)

*V. nutabunda* Yang & Wu, 1994; China (Taiwan)

*V. obaerata* Yang & Wu, 1994; China (Taiwan)

*V. obliqua* Yang & Wu, 1994; China (Taiwan)

*V. parca* Yang & Wu, 1994; China (Taiwan)

*V. pentaprocessusa* Sui & Chen, 2019; China (Yunnan)

*V. shirakii* Matsumura, 1914; China (Taiwan)

*V. stigmata* Matsumura, 1914; China (Guizhou, Hunan, Taiwan, Yunnan)

*V. triprotrusa* Wu & Liang, 2001; China (Guizhou, Yunnan)

*V. umbripennis* Muir, 1914; China (Taiwan)

##### Key to species of the genus *Vekunta* Distant, 1906 from China (male)

**Table:** 

1	Forewing white, yellowish white, pale brown, or with dark markings	**2**
–	Forewing uniformly dark except ScP	**18**
2	Forewing dark brown along costal and anal margins	**3**
–	Forewing not dark brown along costal and anal margins	**6**
3	Mesonotum with lateral areas brownish	***V. fuscolineata***
–	Mesonotum without lateral areas brownish	**4**
4	Anal tube of male symmetrical	**5**
–	Anal tube of male asymmetrical	***V. pentaprocessusa***
5	Gonostyli of male with a bifurcate process at base	***V. bambusana***
–	Gonostyli of male with a bilobed process at base	***V. kotoshonis***
6	Forewing pale brown or most cells suffusedly bordered with pale sepia-brown	**7**
–	Forewing white or yellowish white	**9**
7	Anal tube of male with ventral margin at middle triangularly produced ventrad	***V. makii***
–	Anal tube of male with ventral margin nearly straight	**8**
8	Pygofer of male with symmetrical dorsocaudal processes	***V. lyricen***
–	Pygofer of male with left dorsocaudal process triangularly produced caudad, right dorsocaudal process obtuse	***V. parca***
9	Thorax with propleura with a large dark spot	***V. albipennis***
–	Thorax with propleura not as above	**10**
10	Anal tube of male with apex straight or turned downward but not cephalad	**11**
–	Anal tube of male with apex turned downward then apically cephalad	**16**
11	Anal tube of male with ventral margin at middle nearly straight	**12**
–	Anal tube of male with ventral margin medially produced downward	**15**
12	Periandrium of male without processes	***V. extima***
–	Periandrium of male with two long processes near middle	**13**
13	Periandrium of male with a finger-like process ventrally at base	***V. obaerata***
–	Periandrium of male without a finger-like process ventrally at base	**14**
14	Endosoma of male in right side with a rather large, triangular process at base, tegminating in rod-like process at apex	***V. intermedia***
–	Endosoma of male not as above	***V. obliqua***
15	Periandrium of male with two processes	***V. gracilenta***
–	Periandrium of male with five processes	***V. maculata***
16	Pygofer of male with symmetrical dorsocaudal processes	***V. nivea***
–	Pygofer of male with left dorsocaudal process triangularly produced caudad, right dorsocaudal process obtuse	**17**
17	Endosoma of male with apex reaching to middle of periandrium	***V. commendata***
–	Endosoma of male with apex not reaching to middle of periandrium	***V. nutabunda***
18	Anal tube of male symmetrical	**19**
–	Anal tube of male asymmetrical, strongly inclined to left side	***V. deflecta* sp. nov**.
19	Anal tube of male with apex nearly straight	**20**
–	Anal tube of male with apex curved or turned downward	**25**
20	Pygofer of male with dorsocaudal process longer on right side	***V. asymmetrica***
–	Pygofer of male with symmetrical dorsocaudal processes	**21**
21	Pygofer of male in profile with dorsocaudal processes triangular	**22**
–	Pygofer of male in profile with dorsocaudal processes broadly rounded	**24**
22	Gonostyli of male with an L-shaped process near middle on inner side	***V. triprotrusa***
–	Gonostyli of male without an L-shaped process	**23**
23	Anal tube of male with subapical portion ventral margin extending downward to form an angular projection	***V. fodingshanensis* sp. nov**.
–	Anal tube of male with subapical portion ventral margin nearly straight	***V. malloti***
24	Anal tube of male with ventral margin before middle on each side produced ventrocaudad as a triangular process	***V. memoranda***
–	Anal tube of male with ventral margin at middle angulately produced downward	***V. nigra***
25	Pygofer of male with left dorsocaudal process triangularly produced caudad, right dorsocaudal process obtuse	**26**
–	Not as above	**27**
26	Periandrium of male with a basal process	***V. fera***
–	Periandrium of male without a basal process	***V. umbripennis***
27	Periandrium of male with an elongate, Y-shaped process on left medial subapical region	***V. furcata* sp. nov**.
–	Periandrium of male in left side without processes	***V. stigmata***

Due to the limited availability of female descriptions for many species, the key to the genus presented herein is based primarily on male characters. Five species—*V. atripennis*, *V. botelensis*, *V. diluta*, *V. nigrolineata*, and *V. shirakii*—are known only from females and, therefore, are not included in the key.

### Vekunta deflecta

Taxon classificationAnimaliaHemipteraDerbidae

Yao, Sui & Chen sp. nov.

23ECE8DF-C784-5E2E-8516-9257FFEA2B66

https://zoobank.org/DF467D6F-05E8-447D-8F58-8C51E81611CB

Figs 1, 2, 3, 4, 5, 6, 7, 8, 9, 10, 11, 12, 13

#### Type material.

***Holotype***: China • ♂ (GUGC); Guizhou, Libo; 17.VII.2011; J.-K. Long leg.; light trap (Der11071701). ***Paratypes***: China • 2♂♂ (GUGC); Guizhou, Libo; 16–20.VII.2011; J.-K. Long leg.; sweeping (Der11071702, Der11071703) • 1♂ (GUGC); Guizhou, Libo; 25.VII.2015; Z.-X. Zhou, Y.-Y. Liu leg.; sweeping (Der15072501) • 1♂ (GUGC); Guizhou, Libo; 26.VII.2015; Z.-X. Zhou leg.; sweeping (Der15072601).

#### Measurements.

Body length (including forewing): male 5.48–6.34 mm (*n* = 5); forewing length: male 4.53–5.43 mm (*n* = 5).

#### Description.

***Colouration***. General colour dark brown to nearly black. Head, frons, and clypeus yellow (Figs 1–5). Rostrum (Fig. 4) yellow, with apex fuscous. Gena (Fig. 5) yellow. Eyes (Figs 1–5) black; ocelli yellow. Antennae (Figs 1–5) yellow. Pronotum and mesonotum (Fig. 3) brown. Posterolateral margins of pronotum and tegula yellow (Fig. 3). Forewing (Figs 1, 2) nearly black except for a yellow spot at ScP; veins black. Hindwing subhyaline, brown; veins dark brown. Thorax with ventral areas (Figs 2, 4, 5) yellow. Legs (Fig. 2) yellow. Genital segment dark brown.

***Head and thorax***. Head (Figs 1, 3) with eyes distinctly narrower than pronotum (1:1.71). Vertex (Figs 1, 3) wider at base than length in middle line (1:0.58), apex narrower than base (1:1.50), straightly projecting before eyes; median carina absent; lateral margin distinctly carinate; posterior margin slightly concave. Frons (Fig. 4), near frontoclypeal suture widest; disc concave; lateral margin distinctly carinate; median carina absent. Postclypeus (Fig. 4) with median and lateral carinae. Rostrum (Fig. 4) extending to hind coxae. Antennae (Figs 1–5) short; second antennomere oval; flagellum from apical point. Subantennal processes (Figs 4, 5) small. Eyes (Figs 1–5) semicircular. Ocelli present, adjacent to eyes. Pronotum (Fig. 3) medially short; anterior margin between eyes convex; posterior margin deeply concave; median carina indistinct. Mesonotum (Fig. 3) slightly wider than long, convex; in lateral view raised above vertex; median and lateral carinae weak; posterior end triangularly depressed. Forewing (Fig. 6) narrow; clavus closed; claval veins with a prominent ridge of tubercles; base of costal margin curved inward; costal margin also granulated; RP and MP_1+2_ fused for a short distance; MP_3+4_ single or bifurcated. Hindwing (Fig. 7) with RP reaching apical margin.

***Male terminalia***. Anal tube (Fig. 9) in lateral view elongate, strongly inclined to left; dorsal margin of basal part slightly protruding posteriorly at middle; apical portion curved downward; in dorsal view (Fig. 8), posterior margin concave at middle in profile, segment XI (epiproct and paraproct) sets at basal 1/5. Pygofer (Fig. 9) in lateral view narrowed; dorsocaudal processes of pygofer (Fig. 10) discontinuous at middle in dorsal view, asymmetrical; left process significantly slenderer than right. Gonostyli (Figs 9, 11) asymmetrical; left shorter than right, gradually widening from base to apex; apical margin irregular; inner basal area with a trilobate sac-like process, apical region densely spinose, a slender hook-shaped process caudad to sac-like process. Phallic complex (Figs 12, 13) asymmetrical; periandrium curved, with a finger-like process ventrally at base; right side of periandrium near apex with a tripartite process (P1). Endosoma bearing three lamellate structures (L1, L2, L3) and one elongate process (P2); L1 with a tuberculate sac-like process on inner subapical surface, dorsal margin truncate apically, ventral apical margin sharply pointed; L2 dorsal margin sinuate from base to subapex, apex extended into a hooked process, directed dorsad; L3 with a finger-like process on dorsal margin and an elongate spiniform projection apically; P2 at left side of endosoma base, biapiculate.

#### Remarks.

This species is similar to *V. asymmetrica*, but it can be distinguished by the following features of the male terminalia: anal tube asymmetrical, strongly inclined to the left side (symmetrical in *V. asymmetrica*); periandrium with a finger-like process ventrally at base (without finger-like process in *V. asymmetrica*); right side of periandrium near apex with a tripartite process (without tripartite process in *V. asymmetrica*).

#### Etymology.

The new species name is derived from the Latin word deflecta, referring to the anal tube of male, which is distinctly deflected to the left side.

#### Host plant.

*Bambusa emeiensis* (Poales: Poaceae: Bambusoideae).

#### Distribution.

Known only from the type locality.

### Vekunta fodingshanensis

Taxon classificationAnimaliaHemipteraDerbidae

Yao, Sui & Chen sp. nov.

9DF890AA-4D28-513B-934A-49B495685AE0

https://zoobank.org/21E80D05-F006-484D-881F-ED3BAA7AF590

Figs 14, 15, 16, 17, 18, 19, 20, 21, 22, 23, 24, 25, 26

#### Type material.

***Holotype***: China • ♂ (GUGC); Guizhou, Fodingshan National Nature Reserve; 16.VII.2025; X. Yao, S.-S. Lv and F.-E. Li leg.; sweeping (Der25071601). ***Paratypes***: China • 3♂♂ (GUGC); same data as for holotype (Der25071602, Der25071603, Der25071604).

#### Measurements.

Body length (including forewing): male 5.33–5.40 mm (*n* = 4); forewing length: male 4.40–4.66 mm (*n* = 4).

#### Description.

***Colouration***. General colour yellowish brown (Figs 14–18). Rostrum apex (Fig. 17) fuscous. Eyes (Figs 14–18) black; ocelli light brown. Antennae (Figs 14–18) light brown. Pronotum and mesonotum (Fig. 16) brown. Forewing (Figs 14, 15) fuscous except slightly paler at ScP; veins brown. Hindwing subhyaline, light brown; veins brown.

***Head and thorax***. Head (Figs 14, 16) with eyes distinctly narrower than pronotum (1:1.50). Vertex (Figs 14, 16) wider at base than length in middle line (1:0.65); apex narrower than base (1:1.16), straightly projecting before eyes; median carina absent; lateral margin distinctly carinate; posterior margin slightly concave. Frons (Fig. 17), near frontoclypeal suture widest; disc concave; lateral margin distinctly carinate; median carina absent. Postclypeus (Fig. 17) with median and lateral carinae. Rostrum (Fig. 17) extending to hind coxae. Antennae (Figs 14–18) short; second antennomere oval; flagellum from apical point. Subantennal processes (Figs 17, 18) small. Eyes (Figs 14–18) semicircular. Ocelli present, adjacent to eyes. Median length of pronotum (Fig. 16) short; anterior margin between eyes convex; posterior margin deeply concave; median carina present. Mesonotum (Fig. 16) nearly as long as wide, convex, in lateral view raised above vertex; median and lateral carinae weak; posterior end triangularly depressed. Forewing (Fig. 19) narrow; clavus closed; claval veins with a prominent ridge of tubercles; costal margin curved inward at base and granulated; RP and MP_1+2_ fused for a short distance; MP_3+4_ single. Hindwing (Fig. 20) with RP reaching apical margin.

***Male terminalia***. Anal tube (Fig. 22) in lateral view elongate; subapical portion ventral margin extending downward to form an angular process; in dorsal view (Fig. 21) symmetrical, exhibiting a short median posterior projection on anterior margin, narrowing from base to near midlength, then laterally extended with apex obtusely rounded; segment XI set anterior to midlength. Pygofer (Fig. 22) in lateral view distinctly shorter along dorsal margin than along ventral margin; ventral margin markedly extended posteriorly. Dorsocaudal processes of pygofer (Fig. 23) discontinuous at middle in dorsal view, nearly symmetrical, with left process slightly more slender and elongated than right one. Gonostyli (Figs 22, 24) symmetrical; dorsal and ventral margins subparallel in lateral view; posterior margin slightly concave near dorsal margin; dorsomedial surface near midlength bearing a broad, sac-like process projecting upward to form a robust prominence with slightly pointed apex, followed posteriorly by a hook-shaped process curved mesad. Phallic complex (Figs 25, 26) asymmetrical; periandrium curved, on left dorsobasal region arises a broad lamellar process (L1); L1 gradually narrowing at basal 1/3 into an elongated process extending along ventral margin of periandrium, exceeding midlength and directed leftward. Endosoma comprise four spinose processes (P1–P4) and two complex lamellar structures (L2, L3): P1 from left base; P2 from ventrobasal margin, elongate; P3 dorsally near left midlength (slightly basal); P4 arises ventrally; L2 broad with basal half sinistrally extended and coiled, apex slightly upturned; L3 dextrally positioned, dorsal margin serrated, apex bifurcated.

#### Remarks.

This species is similar to *V. nigra*, but it can be distinguished by the following features of the male terminalia: dorsocaudal processes of pygofer triangular in profile (broadly rounded in *V. nigra*); gonostyli inner surface with a broad sac-like process and a hook-shaped process (a large projection and a small projection indistinct in *V. nigra*); periandrium without paired process at base (with paired process, directed caudad in *V. nigra*).

#### Etymology.

The new species name fodingshanensis refers to the type locality, Fodingshan National Nature Reserve from Guizhou Province.

#### Host plant.

*Bambusa emeiensis* (Poales: Poaceae: Bambusoideae).

#### Distribution.

Known only from the type locality.

### Vekunta furcata

Taxon classificationAnimaliaHemipteraDerbidae

Yao, Sui & Chen sp. nov.

B7BC37F2-4CFC-5651-9934-9C84453329D8

https://zoobank.org/B6B8F1F0-42E3-41BB-84BD-5CC5169445AB

Figs 27, 28, 29, 30, 31, 32, 33, 34, 35, 36, 37, 38, 39

#### Type material.

***Holotype***: China • ♂ (GUGC); Guizhou, Wangmo; 19.VIII.2012; W.-B. Zheng leg.; sweeping; bamboo (Der12081901). ***Paratypes***: China • 1♂ (GUGC); Guizhou, Wangmo; 20.VII.2016; Y.-S. Ding, L.-J. Yang leg.; sweeping (Der16072001) • 1♂ (GUGC); Guizhou, Zhenfeng; 7.X.2017; Y.-J. Sui leg.; sweeping (Der17100701) • 1♂ (GUGC); Guizhou, Ziyun; 3.VII.2019; L. Zhang leg.; sweeping (Der19070301).

#### Measurements.

Body length (including forewing): male 4.45–4.91 mm (*n* = 4); forewing length: male 3.82–4.29 mm (*n* = 4).

#### Description.

***Colouration***. General colour dark brown to nearly black. Head, frons, and clypeus dark brown (Figs 27–31); apical portion of clypeus yellow (Fig. 30). Rostrum (Fig. 30) yellow, with apex fuscous. Gena (Fig. 31) dark brown, with a yellow circumocular ring. Eyes (Figs 27–31) reddish-brown; ocelli yellow. Antennae (Figs 27–31) yellow. Pronotum and mesonotum (Fig. 29) dark brown. Forewing (Figs 27, 28) nearly black, with yellow areas along ScP and RA, separated by a small black zone between these two veins; veins black. Hindwing subhyaline, brown; veins dark brown. Thorax with ventral areas (Figs 28, 30, 31) brown. Legs (Fig. 28) generally yellow. Genital segment dark brown.

***Head and thorax***. Head (Figs 27, 29) with eyes distinctly narrower than pronotum (1:1.64). Vertex (Figs 27, 29) wider at base than length in middle line (1:0.61); apex narrower than base (1:1.39), straightly projecting before eyes; median carina absent; lateral margin distinctly carinate; posterior margin slightly concave. Frons (Fig. 30), near frontoclypeal suture widest; disc concave; lateral margin distinctly carinate; median carina absent. Postclypeus (Fig. 30) with median and lateral carinae. Rostrum (Fig. 30) extending to hind coxae. Antennae (Figs 27–31) short; second antennomere oval; flagellum from apical point. Subantennal processes (Figs 30, 31) small. Eyes (Figs 27–31) semicircular. Ocelli present, adjacent to eyes. Median length of pronotum (Fig. 29) short; anterior margin between eyes convex; posterior margin deeply concave; median carina present. Mesonotum (Fig. 29) slightly wider than long, convex, in lateral view raised above vertex; median carina distinct; lateral carinae absent; posterior end triangularly depressed. Forewing (Fig. 32) narrow; clavus closed; claval veins with a prominent ridge of tubercles; costal margin curved inward at base and granulated; RP and MP_1+2_ fused for a short distance; MP_3+4_ single. Hindwing (Fig. 33) with RP reaching apical margin.

***Male terminalia***. Anal tube (Fig. 35) in lateral view elongate, wider at basal half, slender and curved at apical half; in dorsal view (Fig. 34) symmetrical, slightly wider at base, and exhibiting a short median posterior projection on anterior margin while maintaining nearly parallel lateral margins that extend to apex; segment XI set at 2/5 of basal length. Pygofer (Fig. 35) in lateral view distinctly shorter along dorsal margin than along ventral margin. Dorsocaudal processes of pygofer (Fig. 36) discontinuous at middle in dorsal view, asymmetrical, without extension on left and produced caudad triangularly on right. Gonostyli (Figs 35, 37) symmetrical, slightly broader subbasally in lateral view; apex incurved; dorsal margin bears an irregular, short lamella projecting upward near midlength; inner subbasal area with a finger-like process; middle portion with a dorsally directed, robust hook-shaped process; between the finger-like, hook-shaped process lies a sac-like process, its anterior margin densely covered with spinules; two smaller hook-shaped processes posterior to robust hook-shaped process, one dorsally directed, the other oriented toward apex of gonostyli. Phallic complex (Figs 38, 39) asymmetrical; periandrium curved; on left medial subapical region arises an elongated Y-shaped process (P1); corresponding right position bears a subfalcate, dorsally oriented process (P2) accompanied by a parallel-running membranous strip from whose posteroventral margin projects a lender posterior-directed process (P3). Endosoma comprise two short spines (P4, P5), three lamellar processes (L1, L2, L3), and one membranous structure; P4 from left base of process; P5 from right base. L1 elongate, basal half olive-shaped, bearing serrated dorsal margins; apical half forming a digitiform, cephaloventrally oriented projection; L2 displays irregular contours, terminating in a ventrally directed, digitiform extension at its apex; L3 an acutely pointed, cephalodorsally projecting lamella.

#### Remarks.

This species is similar to *V. umbripennis*, but it can be distinguished by the male terminalia: left dorsocaudal process of pygofer without extension, right one triangularly produced (left one triangularly produced, right one obtuse in *V. umbripennis*); gonostyli with four processes and a sac-like structure (only one small, indistinct process and a sac-like structure in *V. umbripennis*); periandrium with an elongated Y-shaped process on left medial subapical region (absent in *V. umbripennis*).

#### Etymology.

The new species name is derived from the Latin word furcata, meaning “forked” and referring to the Y-shaped process on the periandrium in males.

#### Host plant.

*Bambusa* sp. (Poales, Poaceae, Bambusoideae).

#### Distribution.

Known only from the type locality.

## Discussion

Guizhou Province is in the southwestern hinterland of China and is characterised by its extensive karst landforms that have shaped complex and diverse ecosystems with exceptionally rich biodiversity. Through systematic examination of museum and field collections, three new species of *Vekunta* are described from Guizhou Province, and *V. fuscolineata* is newly recorded for China.

*Vekunta* belongs to the subfamily Derbinae, tribe Cenchreini. At the generic level, *Vekunta* is externally similar to *Goneokara* Muir, 1913 (Yang and Wu 1994), the only other genus of Cenchreini known from China. During field surveys and specimen collection, several specimens of the genus *Goneokara* (unpublished) were obtained. Morphological comparison revealed that the two genera are similar in external appearance. The key diagnostic feature lies in the forewing venation, where RP and MP_1+2_ are fused for a distance, whereas in *Goneokara*, these veins are not fused. Furthermore, this characteristic serves as a stable and important morphological basis distinguishing *Vekunta* from other genera within the tribe.

To date, no infrageneric or species-group classification has been proposed for this genus. Available data indicate that specimens collected from bamboo and those from other hosts share the generic diagnostic characters, without showing any clear morphological divergence. However, the possibility of host-associated morphological differences cannot be ruled out and warrants further investigation. Moreover, some species may not be strictly bamboo-feeding, as occasional records from other plants suggest a broader host range.

Host plant records for *Vekunta* remain scarce. To date, only *Vekunta bri* Löcker, Löcker & Holzinger, 2009 has been documented on shrubs (Löcker et al. 2009). However, based on published literature and field surveys, several species—including *V. bambusana*, *V. triprotrusa*, *V. stigmata*, *V. pentaprocessusa*, *V. fuscolineata*, and three *Vekunta* spp.—have been collected from bamboo (Sui and Chen 2019; Chen and Yang 2023; Chen et al. 2024). Notably, the three new species, *V. deflecta* Yao, Sui & Chen, sp. nov., *V. fodingshanensis* Yao, Sui & Chen, sp. nov., and *V. furcata* Yao, Sui & Chen, sp. nov., described here were also found on bamboo, which further supports the possibility of bamboo serving as important host plants for this genus. Ongoing efforts are focused on collecting host-association data for additional species of *Vekunta*. Nevertheless, a more thorough understanding of these ecological relationships will require further field surveys and more accurate data, which we hope to obtain in future studies.

China is characterised by exceptionally abundant bamboo resources, with Guizhou Province being one of China’s most bamboo-rich regions (Xu et al. 2024). The discovery of three new *Vekunta* species in this area is therefore not unexpected, and further discoveries of new *Vekunta* species in this area are highly anticipated.

## Supplementary Material

XML Treatment for Vekunta

XML Treatment for Vekunta deflecta

XML Treatment for Vekunta fodingshanensis

XML Treatment for Vekunta furcata

## Additional information

### Artificial Intelligence (AI) use

The authors accept full responsibility for the content of the manuscript, including the disclosure of any use of AI.

No AI tools were used in the preparation of this manuscript.

### Author ORCIDs

X. Yao https://orcid.org/0009-0005-1530-3930

Y.-J. Sui https://orcid.org/0000-0001-7514-2984

S.-S. Lv https://orcid.org/0000-0001-5353-5082

J.-K. Long https://orcid.org/0000-0002-4576-9919

Z.-M. Chang https://orcid.org/0000-0002-7247-9706

Z.-Z. Li https://orcid.org/0000-0003-1609-9992

X.-S. Chen https://orcid.org/0000-0001-9801-0343

L. Yang https://orcid.org/0000-0002-7841-5156

### Data availability

All of the data that support the findings of this study are available in the main text.
